# Right-dominant arrhythmogenic cardiomyopathy complicated by platypnea-orthodeoxia syndrome: a novel mechanism of patent foramen Ovale-mediated hypoxaemia: a case report

**DOI:** 10.1093/ehjcr/ytag140

**Published:** 2026-03-03

**Authors:** Xiaoyu Peng, Shuang Xia

**Affiliations:** Department of Cardiology, Guangdong Cardiovascular Institute, Guangdong Provincial People's Hospital, Guangdong Academy of Medical Sciences, Southern Medical University, No. 96, Dongchuan Road, Yuexiu District, Guangzhou, Guangdong 510080, China; Department of Cardiology, Guangdong Cardiovascular Institute, Guangdong Provincial People's Hospital, Guangdong Academy of Medical Sciences, Southern Medical University, No. 96, Dongchuan Road, Yuexiu District, Guangzhou, Guangdong 510080, China

**Keywords:** Platypnea-orthodeoxia syndrome, Right-dominant arrhythmogenic cardiomyopathy, Patent foramen ovale, Atrial flutter, Case report

## Abstract

**Background:**

Platypnea-orthodeoxia syndrome (POS) is a rare but treatable cause of positional hypoxaemia, typically associated with intracardiac shunting. We present the first documented case of POS secondary to patent foramen ovale (PFO) in a patient with right-dominant arrhythmogenic cardiomyopathy (ACM).

**Case summary:**

A 48-year-old male with right-dominant ACM developed progressive dyspnoea and upright hypoxaemia. Workup examinations revealed atrial flutter and PFO with right-to-left shunting. PFO occlusion resolved hypoxaemia, with sustained improvement at 3-year follow-up despite progressive RV dysfunction.

**Discussion:**

This case represents a rare form of POS attributable to the coexistence of PFO and right-dominant ACM. The pathophysiological mechanism underlying POS in this case might be parallel to that observed in right ventricular myocardial infarction or ischaemia in which elevated right-sided intracardiac pressures due to impaired RV output secondary to right heart dysfunction equals or surpasses left heart pressure which created a pressure gradient favourable for right-to-left shunting (RLS) through the PFO in upright position. Based on comprehensive clinical evaluation, PFO closure was ultimately performed.

**Conclusion:**

This case highlights right-dominant ACM as a novel predisposing factor for POS via PFO shunting. The pathophysiological mechanism underlying POS in this case might be parallel to that observed in right ventricular myocardial infarction or ischaemia. PFO closure may be curative for right-dominant ACM-related hypoxaemia and POS.

## Graphical Abstract

**Graphical Abstract:**
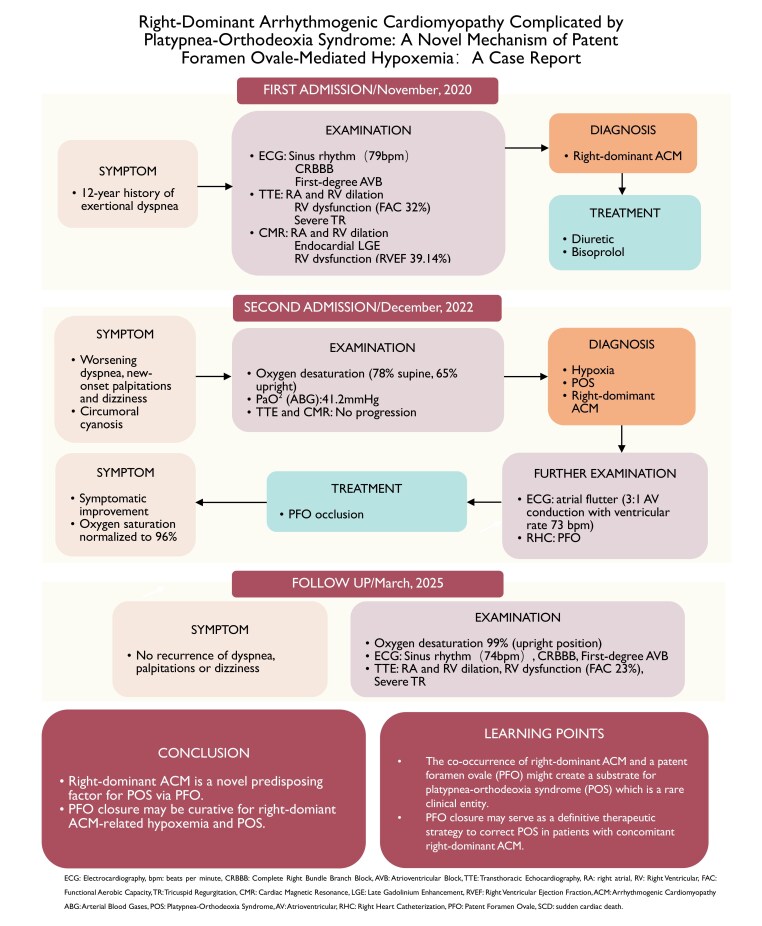

Learning points

- The co-occurrence of right-dominant ACM and a patent foramen ovale (PFO) might create a substrate for platypnea-orthodeoxia syndrome (POS) which is a rare clinical entity.
- PFO closure may serve as a definitive therapeutic strategy to correct POS in patients with concomitant right-dominant ACM.

## Introduction

Platypnea-orthodeoxia syndrome (POS) is an uncommon condition usually characterized by dyspnoea and hypoxaemia in the upright position that improves with recumbency.^1^ Burchell *et al.* described this rare syndrome over half a century ago. The pathophysiology of POS is not completely understood, but the possible pathogenesis necessitates the coexistence of two components:^1^ an interatrial communication defect (e.g. patent foramen ovale, atrial septal defect, or septal aneurysm), pulmonary arteriovenous shunt, or V/Q mismatch and^2^ either structural or haemodynamic alterations enabling orthostatic right-to-left shunting through the defect. Existing POS literature focuses on pulmonary hypertension, post-thoracic surgery, or anatomical variants.^2^ This is the first documented case of POS arising from a patent foramen ovale (PFO) in the setting of right-dominant arrhythmogenic cardiomyopathy (ACM).

## Case presentation

**Figure 1:**
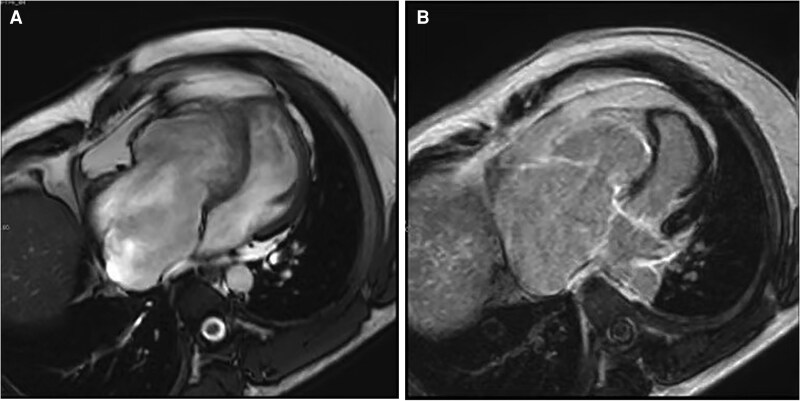
The cardiac magnetic resonance imaging revealed dilated right heart (*A*) and endocardial late gadolinium enhancement in right ventricular (*B*).

A 48-year-old male was admitted to our institution in November 2020 with a 12-year history of recurrent exertional dyspnoea. Electrocardiography revealed sinus rhythm (79bpm) with complete right bundle branch block and first-degree atrioventricular block. Transthoracic echocardiography (TTE) demonstrated right atrial and ventricular dilation, right ventricular (RV) dysfunction (FAC 32%), and severe tricuspid regurgitation (TR). The cardiac magnetic resonance (CMR) imaging revealed endocardial late gadolinium enhancement (LGE), dilated right heart and dyssynchronous motion in RV (RVEF 39.14%) (*Figure 1*). Genetic testing identified a titin (TTN) variant of uncertain significance (VUS). The patient was then diagnosed right-dominant ACM (Padua Criteria, *Table 1*).^3^ Followed electrophysiological study failed to induce ventricular tachycardia or other significant arrhythmias. Given the patient's intermediate-risk profile (2 minor risk factors) and a calculated 5-year risk of 3.0% for life-threatening arrhythmias (>250 bpm VT/VF/SCA) per the ARVC risk calculator,^4^ ICD implantation was deferred (Class IIb recommendation).^5^ Diuretic and bisoprolol were used, and the patient was then discharged.

**Table 1:** Arrhythmogenic cardiomyopathy: diagnostic criteria^3^ and criteria fulfilment in the present case

Category	Right ventricle	
I. Morpho-functional ventricular abnormalities	By echocardiography, CMR or angiography:	
	Major Regional RV akinesia, dyskinesia, or bulging plus one of the following: Global RV dilatation (increase of RV EDV according to the imaging test specific nomograms) − Global RV systolic dysfunction (reduction of RV EF according to the imaging test specific nomograms)	√
	Minor Regional RV akinesia, dyskinesia or aneurysm of RV free wall	
II. Structural myocardial abnormalities	By CE-CMR: Major Transmural LGE (stria pattern) of ≥1 RV region(s) (inlet, outlet, and apex in 2 orthogonal views)	√
	By EMB (limited indications): Major Fibrous replacement of the myocardium in ≥1 sample, with or without fatty tissue	
III. Repolarization abnormalities	Major Inverted T waves in right precordial leads (V1, V2, and V3) or beyond in individuals with complete pubertal development (in the absence of complete RBBB)	
	Minor Inverted T waves in leads V1 and V2 in individuals with completed pubertal development (in the absence of complete RBBB) Inverted T waves in V1, V2, V3 and V4 in individuals with completed pubertal development in the presence of complete RBBB	
IV. Depolarization abnormalities	Minor Epsilon wave (reproducible low-amplitude signals between end of QRS complex to onset of the T wave) in the right precordial leads (V1 to V3) Terminal activation duration of QRS ≥55 ms measured from the nadir of the S wave to the end of the QRS, including R′, in V1, V2, or V3 (in the absence of complete RBBB)	
V. Ventricular arrhythmias	Major Frequent ventricular extrasystoles (N500 per 24 h), non-sustained or sustained ventricular tachycardia of LBBB morphology	
	Minor Frequent ventricular extrasystoles (N500 per 24 h), non-sustained or sustained ventricular tachycardia of LBBB morphology with inferior axis (‘RVOT pattern’)	
VI. Family history/genetics	Major ACM confirmed in a first-degree relative who meets diagnostic criteria ACM confirmed pathologically at autopsy or surgery in a first degree relative Identification of a pathogenic or likely pathogenetic ACM mutation in the patient under evaluation	
	Minor History of ACM in a first-degree relative in whom it is not possible or practical to determine whether the family member meets diagnostic criteria Premature sudden death (b35 years of age) due to suspected ACM in a first-degree relative ACM confirmed pathologically or by diagnostic criteria in a second-degree relative	

ACM, arrhythmogenic cardiomyopathy; EDV, end diastolic volume; EF, ejection fraction; ITF, International Task Force; LBBB, left bundle-branch block; LGE, late gadolinium enhancement; LV, left ventricle; RBBB, right bundle-branch block; RV, right ventricle; RVOT, right ventricular outflow tract.

**Figure 2:**
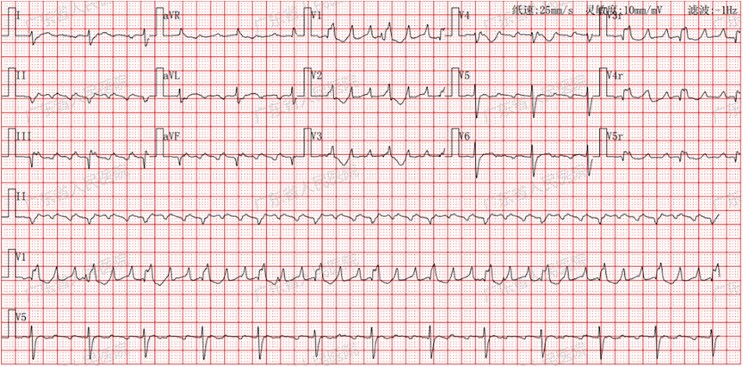
The electrocardiogram revealed atrial flutter (3:1 AV conduction and ventricular rate 73 bpm).

The patient represented with worsening dyspnoea, new-onset palpitations, and dizziness in December 2022. Circumoral cyanosis and notable oxygen desaturation was observed (78% supine, 65% upright). Arterial blood gases drawn on room air revealed PaO^2^ with 41.2 mmHg. The patient was preliminarily diagnosed with right-dominant ACM, hypoxaemia and POS. Further diagnostic workup was arranged to elucidate the aetiology of both hypoxaemia and the underlying POS. The electrocardiogram revealed atrial flutter (3:1 AV conduction and ventricular rate 73 bpm) (*Figure 2*). Chest CT, enhanced cardiac CT and ventilation/perfusion (V/Q) scanning revealed no abnormalities. Follow-up TTE and CMR imaging demonstrated cardiac dysfunction without evidence of disease progression (see Supplementary material online, *Table S2*).

**Figure 3:**
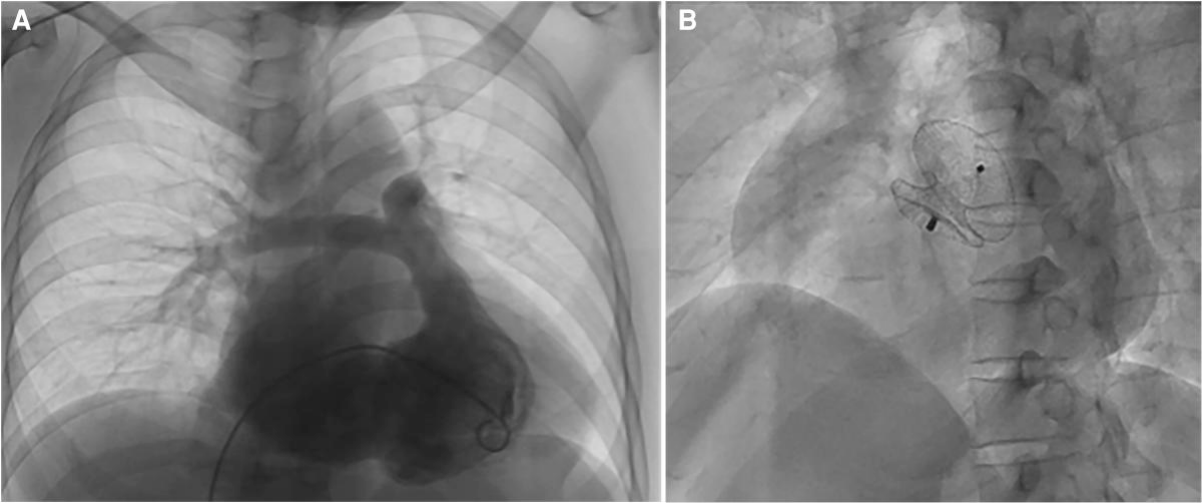
Right heart catheterization revealed a PFO (*A*) which was subsequently occluded (*B*).

Right heart catheterization (RHC) revealed a PFO with mean right atrial pressure (RAP) 7 mmHg, pulmonary capillary wedge pressure (PCWP) 5 mmHg and Qp/Qs 0.66 (see Supplementary material online, *Table S3*). PFO was subsequently occluded (*Figure 3*). Following successful PFO occlusion, the patient experienced symptomatic improvement with peripheral oxygen saturation normalized to 96%, and was subsequently discharged.

## Follow up

At the follow-up evaluation in March 2025, the patient reported no complaints of dyspnoea, palpitations or dizziness and remained clinically stable with oxygen saturation 99% in the upright position. TTE demonstrated post-PFO closure status, along with findings consistent with right-dominant ACM features (RV FAC 23%, severe TR).

## Discussion

To the best of our knowledge, this is the first reported case of POS attributable to concurrent PFO and right-dominant ACM.

POS is an uncommon condition usually characterized by dyspnoea and hypoxaemia in the upright position that improves with recumbency.^1^

An interatrial communication defect, pulmonary arteriovenous shunt, or V/Q mismatch is necessary for POS. Shunting occurs through a PFO in the majority of patients with POS, an atrial septal defect (ASD) in a minority, and rarely in patients with pulmonary arteriovenous malformations.^1^

Two theories have been proposed as aetiologies for right to left shunting (RLS) in PFO patients with POS. The first proposed mechanism is based on altered flow dynamics and streaming of blood from the right to left atrium. This refers to situations of distortion of the atrial septum anatomy or inferior vena cava positioning, resulting in streaming of the inferior vena cava blood through the septal defect. Among these are dilation of the ascending aorta, persistence of the Eustachian valve or Chiari network, lipomatous hypertrophy of the atrial septum, cardiothoracic or abdominal surgeries, pulmonary diseases, kyphoscoliosis, hemidiaphragm paralysis, and chest trauma. The second is haemodynamic based on the pressure gradient between the atria which occurs in hypoxic lung diseases such as (e.g. pulmonary embolism), decreased right-sided compliance (e.g. right ventricular ischaemia) or those associated with high right-sided filling pressures (e.g. pericardial effusion or constrictive pericarditis).^1,6–8^

We postulate that the concomitant presence of PFO and right-dominant ACM accounts for hypoxaemia and POS in this case. The pathophysiological mechanism underlying POS in this case might parallel that observed in right ventricular myocardial infarction or ischaemia in which elevated right-sided intracardiac pressures due to impaired RV output secondary to right heart dysfunction equals or surpasses left heart pressure which created a pressure gradient favourable for RLS through the PFO in upright position.^9,10^

Furthermore, the newly onset atrial flutter (3:1 conduction) may amplified the reversal pressure gradient between right and left heart via tricuspid regurgitation and reduced RV filling efficiency which exacerbated the reversal shunt (SpO_2_ 78%→65% upon standing) (see Supplementary material online, *Videos*). This explains why the patient still developed severe hypoxaemia and POS even without significant worsening of RV function in right-dominant ACM.

The treatment for cardiac POS is percutaneous closure of the interatrial communication, which appears to be safe and efficient, with a low rate of complication.^1^ Notably, this patient developed hypoxaemia even in the supine position, distinguishing this case from previously reported POS patients undergoing closure therapy. While closure therapy could effectively ameliorates right-to-left shunting and hypoxaemia, it may potentially exacerbate right heart failure in this case. Our risk-benefit analysis prioritized correcting life-threatening hypoxaemia (pre-procedure PaO_2_ 41.2 mmHg) over potential right heart decompensation. After comprehensive evaluation, PFO closure was ultimately performed based on the overall clinical assessment. The outcome aligned with our expectations: the patient's hypoxaemia resolved postoperatively without signs of worsened right heart failure during hospitalization. On long-term follow-up, while serial echocardiography showed progressive right ventricular dysfunction, the patient maintained normal oxygen saturation without recurrence of hypoxaemia (see Supplementary material online, *Figure S4*). Notably, the patient did not experience recurrence of atrial flutter, which might be attributed to reduced left atrial pressure and improved oxygenation.

## Conclusion

This is the first report of right-dominant ACM and PFO-mediated POS. The pathophysiological mechanism underlying POS in this case might be parallel to that observed in right ventricular myocardial infarction or ischaemia. Agitated saline echocardiography and/or RHC should be done if shunt suspected. PFO closure may be curative for ARVC-related hypoxaemia and POS, but long-term RV monitoring remains essential.

## Lead author biography


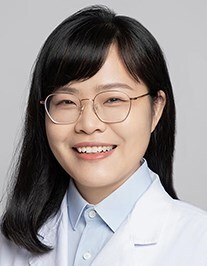


Dr Xiaoyu Peng, MD, earned her doctoral degree from Peking Union Medical College and currently serves as an Attending Physician at Guangdong Provincial People’s Hospital. With over 10 years of experience in clinical practice, teaching, and research, Dr Peng has extensive expertise in the diagnosis and management of common cardiovascular diseases including coronary artery disease, arrhythmia, hypertension, hyperlipidaemia, and heart failure. Her current clinical focus lies in cardiomyopathies and rare/complex cases in cardiology.

## Supplementary Material

ytag140_Supplementary_Data

## Data Availability

Non-identifiable data underlying this article are available to use for all readers.

## Notes

**Consent:** Informed consent and patient details: The authors confirm that written consent for submission and publication of this case report including images and associated text has been obtained from the patient in line with COPE guidance.
