# Supplementary material for: Right-dominant arrhythmogenic cardiomyopathy complicated by platypnea-orthodeoxia syndrome: a novel mechanism of patent foramen Ovale-mediated hypoxaemia: a case report
Source: Eur Heart J Case Rep. 2026 Mar 3;10(3):ytag140. doi: 10.1093/ehjcr/ytag140 (PMC12989647; doi:10.1093/ehjcr/ytag140)
Supplement: ytag140_Supplementary_Data [file ytag140_supplementary_data.zip › Table 2. Parameters from Echocardiography and CMI.docx]

Table 2. Parameters from Echocardiography and Cardiac Magnetic

between the two admissions

| Examination | Parameters | November 2020 | December 2022 |
| --- | --- | --- | --- |
| ECHOCARDIOGRAPHY | FAC | 32% | 38% |
|  | LVEF | 65% | 67% |
| CMR | RVEF | 39.14% | 45% |
|  | RVSV | 48.93ml | 42ml |
|  | LVEF | 48.48% | 56% |
|  | LVSV | 46.64 ml | 51ml |

CMR: cardiac magnetic resonance, FAC: fractional area change, LVEF: left ventricular ejection fraction, RVEF: right ventricular ejection fraction, RVSV: right ventricular stroke volume, LVSV: left ventricular stroke volume.
