# Supplementary material for: Right-dominant arrhythmogenic cardiomyopathy complicated by platypnea-orthodeoxia syndrome: a novel mechanism of patent foramen Ovale-mediated hypoxaemia: a case report
Source: Eur Heart J Case Rep. 2026 Mar 3;10(3):ytag140. doi: 10.1093/ehjcr/ytag140 (PMC12989647; doi:10.1093/ehjcr/ytag140)
Supplement: ytag140_Supplementary_Data [file ytag140_supplementary_data.zip › Table 3. Right Heart Catheterization Findings.docx]

Table 3. Pre-PFO Closure Hemodynamics: Right Heart Catheterization Findings

| Parameters | Mean RAP | Mean PAP | PCWP | PVR | Qp: Qs |
| --- | --- | --- | --- | --- | --- |
| Value | 7mmHg | 10mmHg | 5mmHg | 5.79wood | 0.66 |

RAP: right atrial pressure, PAP: pulmonary arterial pressure, PCWP: pulmonary capillary wedge pressure, PVR: pulmonary vascular resistance.
