# Supplementary figures and images for: Right-dominant arrhythmogenic cardiomyopathy complicated by platypnea-orthodeoxia syndrome: a novel mechanism of patent foramen Ovale-mediated hypoxaemia: a case report
Source: Eur Heart J Case Rep. 2026 Mar 3;10(3):ytag140. doi: 10.1093/ehjcr/ytag140 (PMC12989647; doi:10.1093/ehjcr/ytag140)

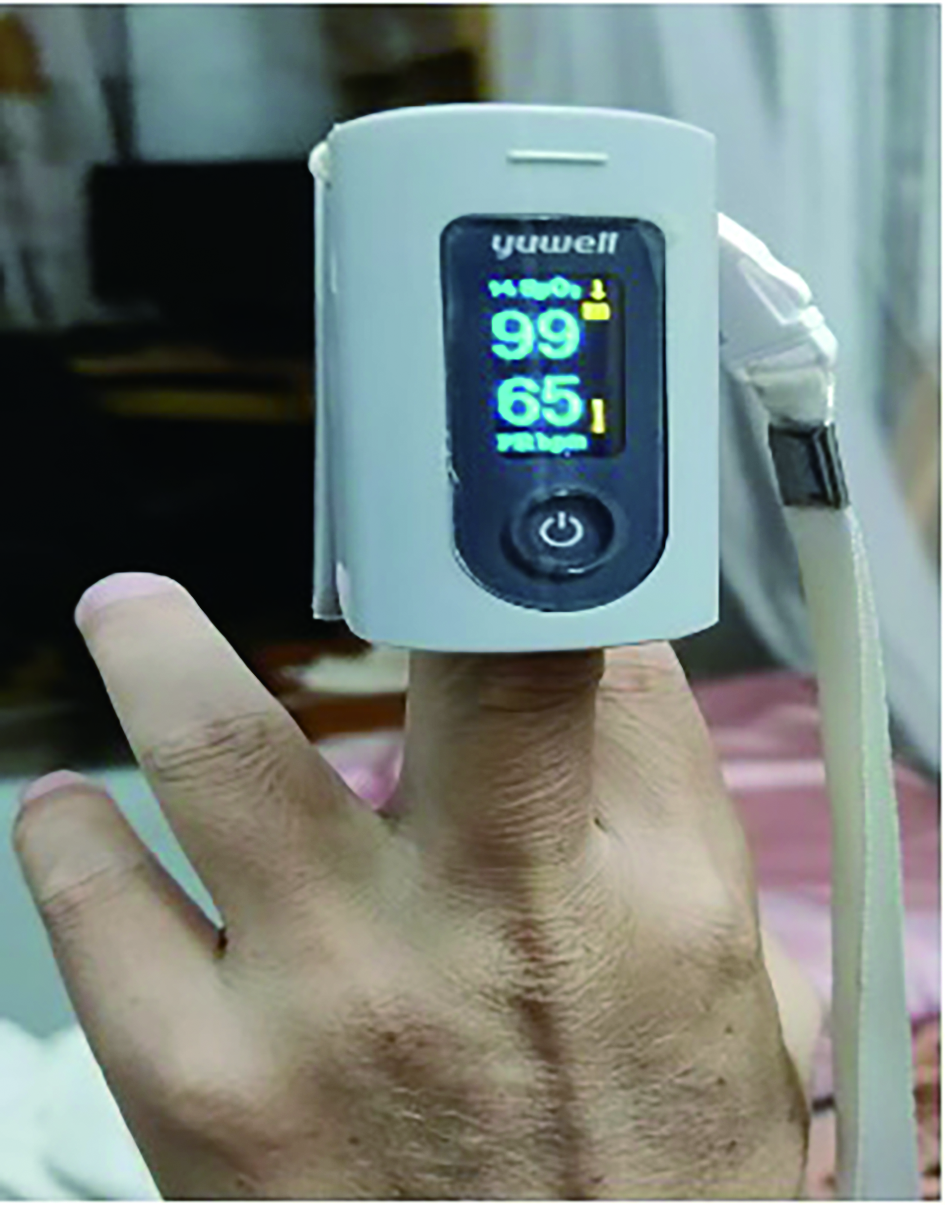

Supplement: ytag140_Supplementary_Data [file ytag140_supplementary_data.zip › Figure4A.tif]

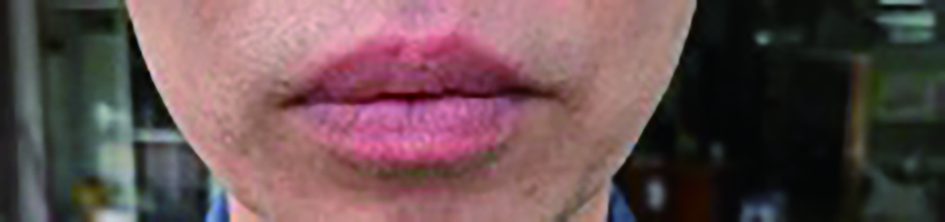

Supplement: ytag140_Supplementary_Data [file ytag140_supplementary_data.zip › Figure4B.tif]
